# Microstructural Design for Improving Ductility of An Initially Brittle Refractory High Entropy Alloy

**DOI:** 10.1038/s41598-018-27144-3

**Published:** 2018-06-11

**Authors:** V. Soni, O. N. Senkov, B. Gwalani, D. B. Miracle, R. Banerjee

**Affiliations:** 1 Department of Materials Science and Engineering, University of North Texas, Denton, 76207 Texas USA; 2 Advanced Materials and Manufacturing Processes Institute, University of North Texas, Denton, 76207 Texas USA; 3 Air Force Research Laboratory, Materials and Manufacturing Directorate, Wright-Patterson AFB, OH-45433 USA; 4 UES Inc, 4401 Dayton-Xenia Road, Beavercreek, OH USA

## Abstract

Typically, refractory high-entropy alloys (RHEAs), comprising a two-phase ordered B2 + BCC microstructure, exhibit extraordinarily high yield strengths, but poor ductility at room temperature, limiting their engineering application. The poor ductility is attributed to the continuous matrix being the ordered B2 phase in these alloys. This paper presents a novel approach to microstructural engineering of RHEAs to form an “inverted” BCC + B2 microstructure with discrete B2 precipitates dispersed within a continuous BCC matrix, resulting in improved room temperature compressive ductility, while maintaining high yield strength at both room and elevated temperature.

## Introduction

Refractory high-entropy alloys (RHEAs) are a relatively new class of multicomponent materials that are based on several refractory metals, but may also contain other, generally low density, elements and typically have a body centered cubic (BCC) crystal structure^1–3^. These alloys have recently received much attention because of their attractive combination of properties not achievable by other classes of metallic alloys. For example, the first two RHEAs, NbMoTaW and NbMoTaVW, showed weak temperature dependence of yield strength in the temperature range from 600 °C to 1600 °C, with yield strength above 400 MPa at 1600 °C^4,5^. Unfortunately, these two alloys had high density (>12 g/cm^3^) and further developments aimed at reducing the alloy density while keeping superior high-temperature properties. In the second generation RHEAs, high-density Ta and W were replaced with lower density refractory elements, such as Cr, Mo, Nb, V and Zr, and low density Al and Ti were added^6–13^. This resulted in RHEAs with lower densities and high-temperature strengths better than the properties of Ni-based superalloys and Fe-based steels^14^. With the exception of the equimolar HfNbTaTiZr^15–19^ and some of its derived compositions^20,21^ most of the reported RHEAs have poor room temperature compressive plasticity, which makes them difficult to process and limits their engineering applications. First-principles calculations showed that alloying intrinsically brittle Mo and W with subgroup IV or V transition metals can make them intrinsically ductile^22^. This composition-induced brittle to ductile transition was explained by changes in the electronic structure, which induced Jahn-Teller distortions and transitioned the elastic instability mode from tensile to shear failure^22^. Extending this theory to RHEAs, Sheikh, *et al*.^21^ found that single-phase BCC RHEAs consisting of subgroup IV, V and VI metals are intrinsically ductile if the valence electron concentration (VEC) is less than 4.5 and intrinsically brittle if VEC ≥4.6.

Huang, *et al*.^23^ and Lilensten, *et al*.^24^ used a “metastability-engineering” approach, which is similar to well-known transformation-induced plasticity (TRIP), to improve tensile ductility of BCC RHEAs by tailoring the stability of constituent phases. Transformation-induced increase in tensile strain and work-hardening capability were successfully achieved by inducing formation of a stronger HCP phase in a strain-localization region of tensile-tested Ta_0.5_HfZrTi^23^ or α” martensite in HfNb_0.18_Ta_0.18_Ti_1.27_Zr^24^. Strain-induced precipitation of the second phase inside the metastable BCC matrix caused strain hardening and slowed strain localization, thus increasing elongation. Similar approaches to enhance uniform tensile ductility resulting from deformation-induced phase transformation were discussed in steels^25,26^, BCC based titanium alloys^27,28^, and FCC-based HEAs^29^. Unfortunately, this method is only applicable to intrinsically ductile RHEAs that show low tensile ductility due to rapid strain localization and necking. It cannot be applied to inherently brittle RHEAs that fracture without strain localization/necking and, often, without any macroscopic strain.

Recently, several Al-containing RHEAs were reported^9,30,31^ to have a characteristic superalloy-like microstructure, consisting of cuboidal BCC nano-scale precipitates within a coherent B2 matrix, resembling the γ(fcc) + γ’(ordered L1_2_ precipitates) microstructure exhibited by many currently used nickel base superalloys. Although they showed exceptionally good strength at both room and elevated temperatures, substantially exceeding those of single-phase BCC RHEAs, these novel two-phase RHEAs have very limited room temperature compressive ductility, which can be explained by the inherent brittleness of the ordered B2 matrix phase^32^. Unfortunately, the approaches for improving ductility discussed above cannot be applied to this class of Al-containing RHEAs.

The present work is the first demonstration of enhancing the ductility of high-strength BCC + B2 two-phase RHEAs by controlling their microstructure. For this, Al_0.5_NbTa_0.8_Ti_1.5_V_0.2_Zr was selected, based on its low density (7.4 g/cm^3^) and previous reports of excellent room and high temperature yield strength^9^. This alloy was cast, hot isostatically pressed (HIPed) and then homogenized at 1200 °C for 24hrs followed by slow cooling (10 °C/min) to room temperature. The resultant microstructure consists of two BCC phases (one of which is likely ordered, but this was not previously proven^9^) with very similar lattice parameters that form a very fine, inter-woven baskteweave-like, nano-phase structure^9^. Subsequently, this will be referred as Condition (1). The alloy has a room-temperature yield strength of 2035 MPa but only 4.5% compression strain before fracture in Condition (1). The present study focuses on improving the ductility of this alloy by tuning the microstructure, while maintaining its high yield strength.

The alloy in Condition (1) was solutionized at 1400 °C for 20 min followed by water quenching to achieve a single-phase microstructure. This will be referred to as Condition (2). The alloy in Condition (2) was then annealed at 600 °C for 120 hrs and water-quenched (subsequently referred to as Condition (3)) to possibly develop a two-phase BCC + B2 microstructure.

## Results

### Microstructure Characterization

**Figure 1:**
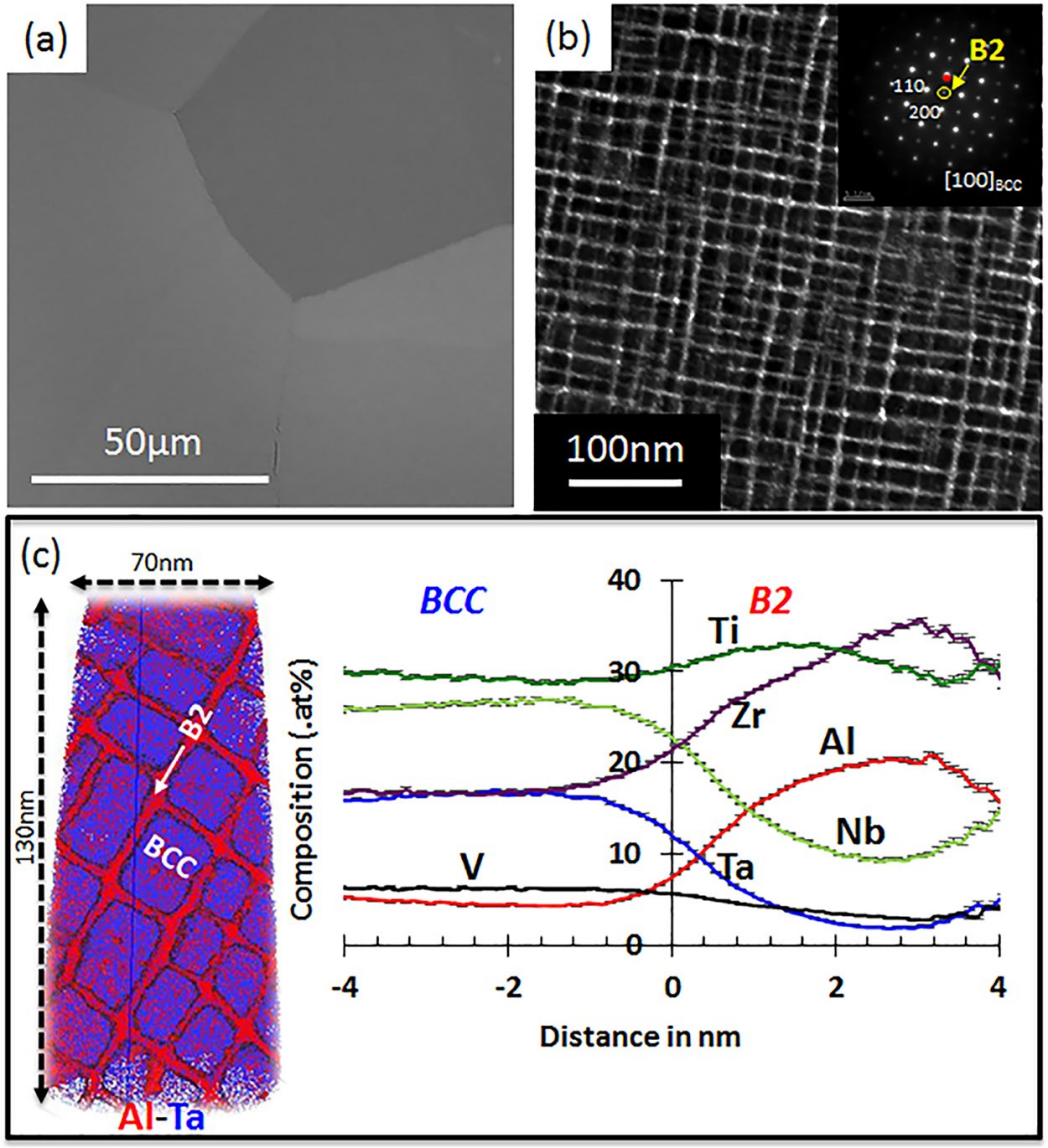
Microstructure of Al_0.5_NbTa_0.8_Ti_1.5_V_0.2_Zr in the cast, hot iso-statically pressed (HIPed) and homogenized (1200 °C/24 hr/slow cool) condition (Condition 1): (**a**) BSE image of coarse equiaxed grains; (**b**) TEM dark-field image showing the continuous channels of an ordered B2 phase (bright) and cuboidal precipitates of a disordered BCC phase (dark) (<100>_BCC_ SADP shown as inset); (**c**) APT re-construction of Al- (red) and Ta- (blue) rich regions (left) and compositional changes (proximity histogram) across a BCC-B2 interface (right).

Condition (1) was studied here in greater detail using scanning electron microscopy (SEM), transmission electron microscopy (TEM) and atom probe tomography (APT). The Condition (1) microstructure is summarized in Fig. 1(a–c). Annealing at 1200 °C resulted in large, equiaxed grains (grain size = 100 µm^9^) as seen in the backscattered electron (BSE) SEM image in Fig. 1(a). A dark-field TEM image, acquired using a {001} superlattice reflection of the B2 phase, is shown in Fig. 1(b). The [001] zone axis electron diffraction pattern is shown as an inset in the same figure. This dark-field TEM image of Condition (1) revealed a highly-refined microstructure consisting of a two-phase mixture. The highlighted brighter regions, forming the continuous matrix, correspond to the ordered B2 phase while the darker discrete pockets correspond to the disordered BCC phase. The edge-to-edge length of the precipitates (disordered BCC) is ~20 nm and the thickness of the channels (ordered B2) is ~2 nm. The precipitates had a very narrow size distribution and were arranged in regular rows along < 001 > direction.

The three-dimensional (3D) distribution of the B2 and BCC phases, and the elemental partitioning across the interface, was studied using atom probe tomography (APT). An example of the reconstructed APT dataset is shown in Fig. 1(c), depicting the raw ion map using Al (red) and Ta (blue) ions. Clearly, there is strong compositional partitioning of the constituent elements within the B2 and BCC phases. The composition profiles for the different elements were plotted using a proximity histogram approach^33^. These profiles are constructed by delineating the B2/BCC interface using an iso-concentration surface of Al = 10.5 at%. The B2 phase (highlighted in red in the reconstruction map) is rich in Al and Zr whereas the BCC phase is rich in Nb and Ta (Fig. 1(c)). The approximate compositions of the two phases are: BCC: 5Al-27Nb-18Ta-11Zr-33Ti-6V (at%) and B2: 20Al-10Nb-4Ta-31Zr-31Ti-4V (at%).

**Figure 2:**
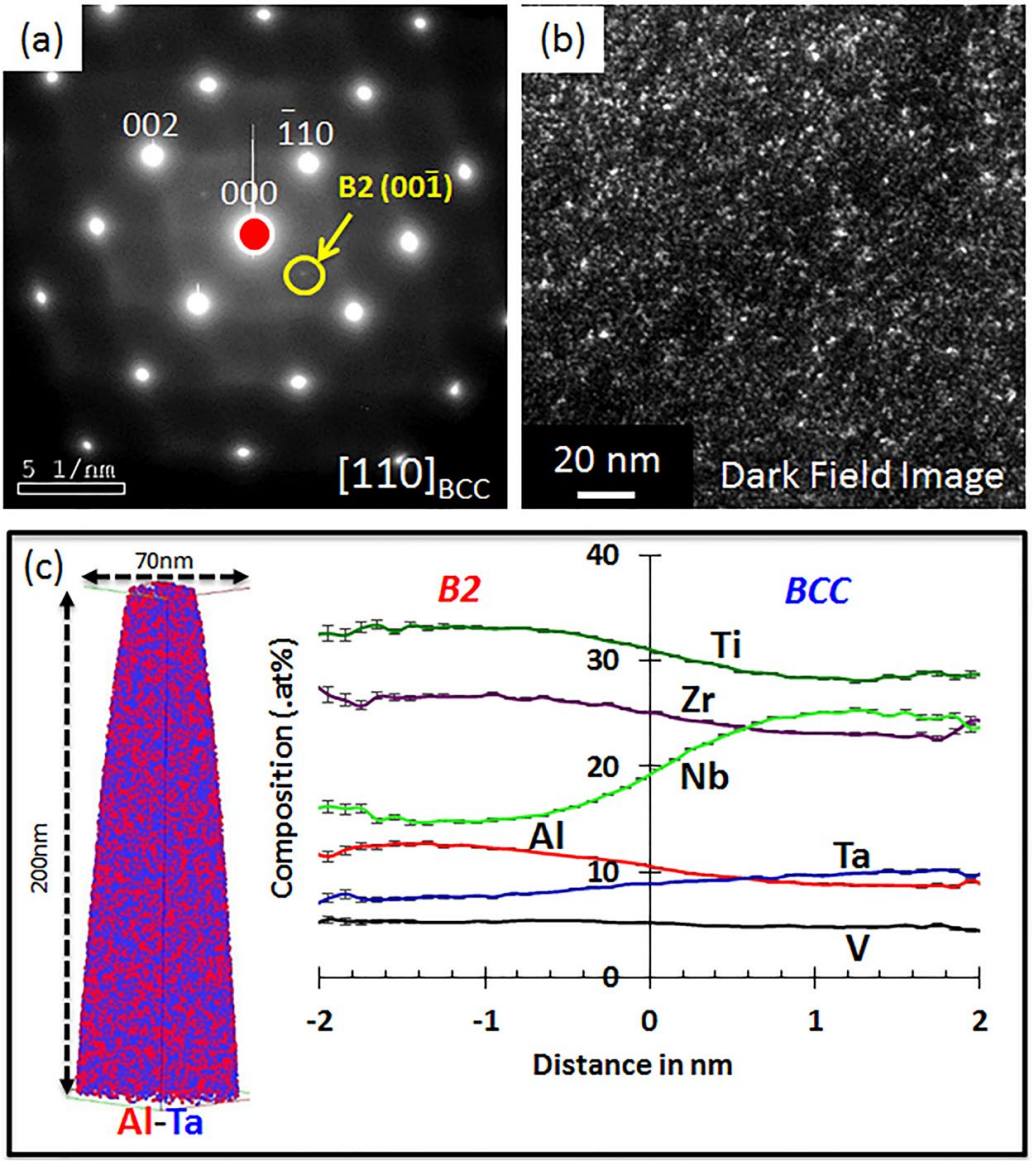
Microstructure of Al_0.5_NbTa_0.8_Ti_1.5_V_0.2_Zr in Condition (1) followed by additional annealing at 1400 °C for 20 min and water quenching (Condition 2): (**a**) Selected area diffraction pattern showing a BCC crystal lattice with very weak B2 super-lattice reflections; (**b**) Dark field image taken from the weak B2 superlattice reflection shown in (a); (**c**) APT reconstruction of Al (red) and Ta (blue) rich regions (left), and compositional changes across BCC/B2 interfaces using a proxygram analysis (right).

The microstructure of the alloy in Condition (2) was substantially different from Condition (1). The average grain size in this condition is ~150 µm. Figure 2(a) shows a selected area electron diffraction pattern from the alloy in Condition (2), which can be indexed as the <011>_BCC_ zone axis. Additionally, careful analysis of the <011>_BCC_ zone axis (Fig. 2(a)) revealed extremely weak {100} B2 superlattice reflections, indicating that this BCC-based phase has weak B2-type ordering. A dark-field TEM image, recorded from one of these {100} B2 reflections, shown in Fig. 2(b), clearly shows highly refined nanometer scale ordered B2 regions dispersed within a BCC matrix. Atom probe tomography was used to further investigate this condition. A raw ion map consisting of Al and Ta ions is shown in Fig. 2(c). Despite the absence of any sharp demarcation between compositionally distinct phases, there appears to be a small degree of inhomogeneity (clustering) in this compositional map. This inhomogeneity has been captured by plotting composition profiles (proximity histogram analysis) for the different constituent elements across an artificially created interface using an iso-concentration surface of Nb = 20at%, as shown in Fig. 2(c). This compositional analysis indicates an early stage of phase separation into a co-continuous mixture of Al, Zr and Ti rich regions interspersed with Nb and Ta rich regions (Fig. 2(c)). This weak partitioning suggests that the alloy was in the single BCC phase field at the solution treatment temperature of 1400 °C, and the early stages of composition partitioning, via fluctuations, occurred during the quench. The partitioning also suggests the existence of a miscibility gap in this HEA composition, similar to previously studied AlMo_0.5_NbTa_0.5_TiZr^32^. The final microstructure in case of Condition (2) can be described as highly refined, nanometer-scale mixture of ordered B2 regions within a BCC matrix.

**Figure 3:**
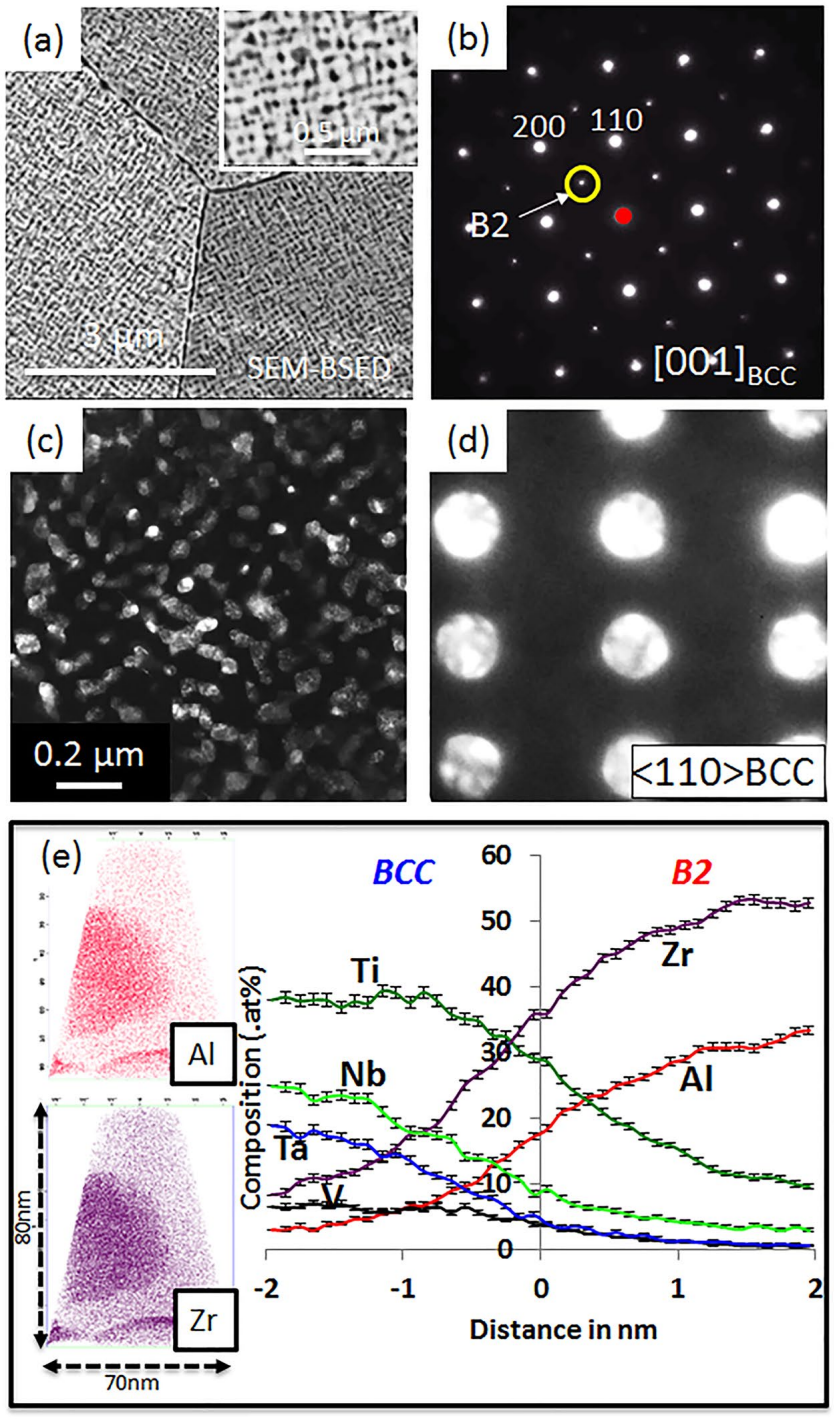
Microstructure of Al_0.5_NbTa_0.8_Ti_1.5_V_0.2_Zr in Condition (2) followed by additional annealing at 600 °C for 120 hrs followed by a water quench (Condition 3): (**a**) Back-scattered SEM image; (**b**) Selected area diffraction pattern of <001>_BCC_ zone axis showing B2 superlattice reflections; (**c**) Dark field TEM image taken from a B2 superlattice spot in <001>_BCC_ zone axis; (**d**) micro-diffraction patterns from BCC phase confirming the presence of disordered BCC phase; (**e**) APT ion maps showing partitioning of elements (left) and compositional profiles across the BCC/B2 interface using proxigram analysis (right).

Condition (3) exhibits a typical superalloy type microstructure as seen in the backscatter SEM image in Fig. 3(a). The average grain size in this condition is ~150 µm. The bright, continuous matrix phase has homogeneously-distributed second phase precipitates (darker contrast) arranged in a checkerboard-like pattern. A TEM dark field image (Fig. 3(c)), obtained from the {100} B2 superlattice spot in the <001>_BCC_ zone axis (Fig. 3(b)), revealed that the discrete precipitates are the ordered B2 phase, while the continuous matrix phase is the disordered BCC phase. The lack of {100} B2 superlattice reflections in the micro-diffraction pattern from the [011]_BCC_ zone axis, recorded from the continuous matrix phase (Fig. 3(d)), indicates that the matrix phase is likely to be a disordered BCC phase. The size scale of the discrete B2 precipitates is ~50 nm. APT results from this microstructure are shown in Fig. 3(e), and the proximity histogram analysis (using an iso-concentration surface of Al = 20 at%) reveals that Al and Zr partition to the B2 phase whereas the BCC phase is rich in the other elements. Based on the proxigram analysis the approximate compositions of the B2 and BCC phases are as follows: B2: 33Al-2Nb-11Ti-54Zr (at%) and BCC: 2Al-25Nb-12Ta-37Ti-8V-16Zr (at%).

### Mechanical Properties

The average Vickers microhardness values with standard deviations, of Conditions (1), (2) and (3), are 562 ± 16 HV, 405 ± 11 HV and 475 ± 12 HV, respectively. The highest hardness was found in Condition (1) due to its fine-scale B2 (hard) + BCC decomposed microstructure, as compared to the single-phase weakly ordered BCC microstructure in Condition (2). Intermediate hardness was observed in Condition (3) due to the presence of discrete B2 precipitates within the continuous BCC matrix, since the scale of the two-phase BCC + B2 microstructure in Condition (3) is substantially coarser than Condition (1).

**Figure 4:**
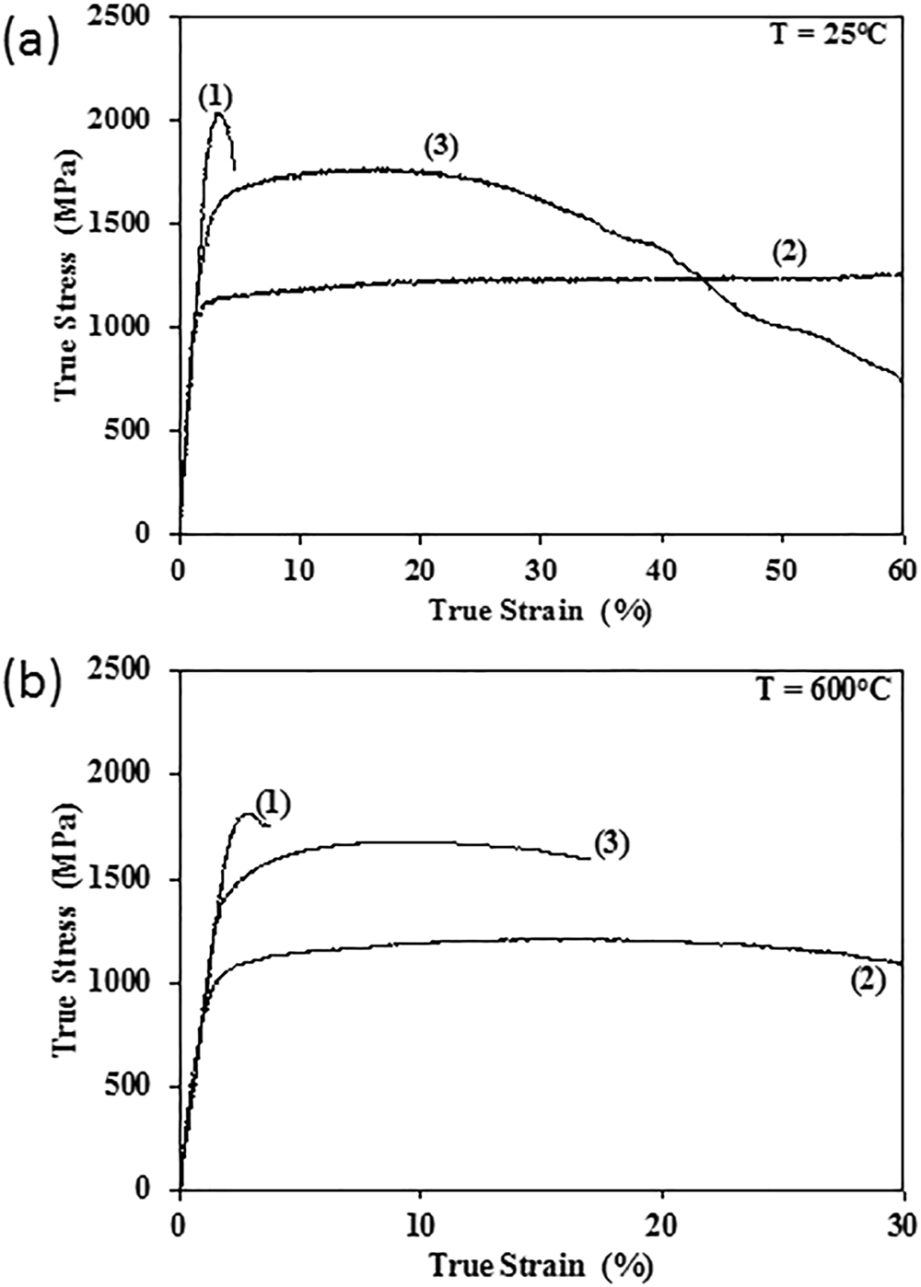
Compressive true stress vs. true strain deformation behavior of the Al_0.5_NbTa_0.8_Ti_1.5_V_0.2_Zr alloy samples for three different heat treatment conditions: (1) – cast plus HIP followed by annealing at 1200 °C for 24 h and slow cooling, (2) – Condition (1) plus additional annealing at 1400 °C for 20 min and water quenching, and (3) – Condition (2) plus additional annealing at 600 °C for 120 hrs and water quenching. The testing temperatures are (**a**) T = 25 °C, (**b**) T = 600 °C.

Compression properties (true stress vs. true strain behavior) of this RHEA were strongly dependent on the microstructure (Fig. 4, Table 1). In Condition (1) the alloy had high room temperature (RT) strength (yield stress *σ*_0.2_ = 2032 MPa, true peak stress *σ*_p_ = 2035 MPa) but showed a true compressive fracture strain *ε*_f_ of only 4.7%. In Condition (2), the alloy was very ductile at room temperature and did not show any evidence of fracture even after a true strain of 60%, with a noticeably lower yield stress (*σ*_0.2_ = 1065 MPa). The alloy in Condition (2) showed continuous strengthening after yielding at a strain hardening rate dσ/dε ≈ 118 MPa. The brittle nature of Condition (1) is due to the presence of continuous B2 channels, whereas Condition (2) is largely a single-phase BCC alloy with a small degree compositional fluctuations, and thus has more ductility. Condition (3) provided an intermediate room temperature yield stress (*σ*_0.2_ = 1345 MPa) and showed noticeable hardening, with the average hardening rate of dσ/dε ≈ 1220 MPa, until *σ*_p_ = 1772 MPa was reached at the peak true compressive strain *ε*_p_ = 16.9%. This hardening rate is considerably higher than that observed in Condition (2). After that, a strain softening stage was observed (Fig. 4(a)). First cracks were detected at *ε*_f_ = 38% and *σ*_f_ = 1410 MPa, but the cracks propagated slowly and fracture in Condition (3) did not occur abruptly, as in Condition (1), but progressively, and deformation was stopped at a true stress of 665 MPa and ε = 60%.

**Table 1:** Compression properties (yield stress, σ_0.2_, peak stress, σ_p_, true strain *ε*_p_ at peak stress, and fracture strain, *ε*_f_) of Al_0.5_NbTa_0.8_Ti_1.5_V_0.2_Zr in different heat treatment conditions.

Alloy Condition	T, °C	*σ*_0.2_, MPa	*σ*_p_, MPa	*ε*_p_, %	*ε*_f_, %
Condition (1)	25	2032	2035	3.5	4.7
	600	1774	1814	2.8	3.8
Condition (2)	25	1065	1250	38	>60
	600	975	1274	63	>60
Condition (3)	25	1345	1772	16.9	38
	600	1423	1682	16.2	16.2

Microstructure showed the same influence on strength as for hardness. Condition (2) was the weakest, as it is best characterized as a single-phase solid solution with no strengthening precipitates. Conditions (1) and (3) are both characterized as precipitation–hardened alloys resembling the microstructure of a γ (fcc) + γ’ (ordered L1_2_) Ni-base superalloy. However, Condition (1) is stronger (but less ductile) than Condition (3) since the harder B2 phase is continuous in Condition (1) while the softer BCC phase is continuous in Condition (3). Further, the scale of the two-phase BCC + B2 microstructure in Condition (3) is substantially coarser than Condition (1).

High temperature compression testing was carried out at 600 °C for all three conditions, and the results are shown in Fig. 4(b). The alloy in Condition (1) exhibited a high yield stress (*σ*_0.2_ = 1774 MPa) but low compressive plasticity (*ε*_f_ = 3.8%), while the yield stress was lower in Condition (3) (*σ*_0.2_ = 1423 MPa) but the compressive plasticity was substantially higher (*ε*_f_ = 16.2%). In both conditions, fracture occurred catastrophically. Condition (2) exhibited the lowest yield stress, *σ*_0.2_ = 975 MPa, but very high compressive plasticity with *ε*_f_ > 60%. Condition (3) had a slightly higher yield stress and lower compressive plasticity at 600 °C than at 25 °C. The reasons for this unusual behavior have important implications for the development of this alloy family for high temperature applications and are discussed in the following section.

Overall, Condition (1) has the highest strength; Condition (2) has the highest compressive ductility; while Condition (3) provides a combination of high yield stress and good ductility.

## Discussion

Important details of the phase stabilities and phase transformations in this alloy remain uncertain. Based on the results, it can be assumed here that the alloy is a disordered BCC single-phase structure at 1400 °C, and that the fine-scale composition modulations observed in APT (Fig. 2(c)) develop during the quench. However, it is possible that the alloy may be undergoing phase separation at 1400 °C. These possibilities need to be studied in future work. Condition (1), the initial “as-processed” condition, exhibited a “superalloy-like” microstructure consisting of cuboidal pockets of BCC within a continuous matrix of the ordered B2 phase. Isothermal annealing of Condition (1) at 1400 °C for 20 mins resulted in disordering of the microstructure to form a single BCC phase, followed by early stages of B2 ordering during water-quenching, resulting in Condition (2). Similar experiments have been carried out where Condition (1) samples were isothermally annealed at 1200 °C and water-quenched, with the resulting microstructure being identical to Condition (2). These results indicate that the “superalloy-like” microstructure in Condition (1) formed during slow-cooling at 10 °C/min from 1200 °C. However, this “superalloy-like” microstructure in Condition (1) changes substantially during a subsequent isothermal anneal at 600 °C for 120 h, resulting in discrete B2 precipitates within a continuous BCC matrix (Condition (3)). This has been confirmed by isothermally annealing a sample of Condition (1) directly at 600 °C for 120 h, and the resultant microstructure is shown in the supplementary Fig. S1. This Fig. S1 confirms that the microstructure developing after directly annealing Condition (1) is the same as Condition (3) which forms after annealing Condition (2). Therefore, it appears that the as-processed Condition (1), exhibiting a “superalloy-like” microstructure, with cuboidal pockets of BCC within a continuous matrix of the ordered B2 phase, was not an equilibrium microstructure for this alloy at 600 °C. Rather the phase fractions of B2 and BCC phases are far-from equilibrium, and reflected an early stage of decomposition. Consequently, long-term isothermal anneal at 600 °C results in an increase in the B2 phase fraction accompanied by a reduction in the BCC phase fraction. This change in phase fractions is presumably accomplished via progressive increase in the width of the B2 channels (starting from Condition (1)), eventually leading to breaking up of these channels into discrete B2 precipitates.

The experimental results presented in this paper clearly show that Condition (1) has the highest strength; Condition (2) has the highest compressive ductility; while Condition (3) provides a combination of high yield stress and good ductility. These differences in compressive properties can be broadly rationalized based on the respective microstructures and phases observed in these three conditions. Thus, in case of Condition (1), the “superalloy-like” microstructure consists of a continuous ordered B2 matrix, interspersed with a regular array of disordered cuboidal BCC precipitates. While the B2 matrix provides a very high strength to this condition, it suffers from rather poor ductility due to its ordered nature. Condition (2) exhibits a microstructure consisting of a highly refined, nanometer-scale mixture of ordered B2 regions within a BCC matrix. Consequently, this condition exhibits a very high compressive ductility while maintaining a reasonably high strength (>1 GPa), since the matrix is disordered BCC and is strengthened by the refined B2 precipitates. Condition (3) consists of a disordered continuous BCC matrix with a higher phase fraction of coarser B2 precipitates, as compared to Condition (1). While the higher B2 phase fraction is beneficial for strength, the continuous BCC matrix provides good compressive ductility. Despite the higher B2 phase fraction in Condition (3) as compared to Condition (1), the change in the continuous phase from BCC in Condition (3) to B2 in Condition (1) is responsible for the higher compressive strength in Condition (1).

The main goal of the present work was to improve the room temperature ductility of an initially brittle RHEA through microstructure modifications by thermal treatment. The goal was successfully achieved and an improved balance of high strength and good compressive ductility was demonstrated by “inverting” the microstructure from a continuous B2 matrix with discrete BCC precipitates to one consisting of a continuous BCC matrix with discrete B2 precipitates. The details of the strengthening mechanisms in these microstructures are beyond of the scope of the present study. However, it can be speculated that strong element bonding in the B2 matrix is responsible for high strength (and, unfortunately, brittleness) of Condition (1); while in the case of Conditions (2) and (3), precipitation strengthening from B2 precipitates and dislocation mobility in BCC matrix are likely to play a substantial role. Classical precipitation hardening models^34^ provide guidance on material parameters that must be quantified to predict the level of strengthening in the present alloys, and more recent models specifically address the influence of coherent B2 precipitates within a disordered BCC matrix in HEAs^35^ and in maraging steels^36^. However, the application of these precipitation strengthening models requires knowledge of a number of parameters, including: the degree of short-range ordering as a function of thermal treatment; the size and spacing of the short-range ordered regions; the lattice misfit between the BCC and B2 phases (requiring high resolution X-ray diffraction measurements); the elastic properties of the BCC and B2 phases; interfacial and fault energies; and the volume fraction of the B2 precipitates. While simple approximations may suffice for some of these parameters in less complicated alloys, these parameters can vary a great deal in the current RHEA, and the approximations are likely to give large uncertainties. Additional scientific studies are therefore required to establish the influence of concentrated alloy additions (especially to the B2 phase) on the multitude of parameters that can influence precipitation strengthening in these complex RHEAs.

The strength of metals almost always decreases with increasing temperature, and so the observation in the present work that the yield strength is higher at 600 °C than at RT for Condition (3) is unusual. The magnitude of the increase shown in the present work is about 6%, which is significantly larger than a typical sample-to-sample experimental uncertainty of ± 1%.

The data needed to establish the mechanism responsible for the anomalous strengthening measured for Condition (3) are difficult to obtain and are beyond the scope of the present work. Nevertheless, it is possible to speculate on the potential mechanisms. The best-known example of anomalous strengthening is for the long-range ordered intermetallic compound, Ni_3_Al, where cross-slip occurs between two competing glide systems, <011>{111} and <011>{100). Sessile kinks produced by this cross-slip strengthen the alloy with increasing temperature, until a temperature is reached where the sessile segments become glissile and strength then decreases with further increases in temperature. Such a mechanism is conceivable in the present alloy, since the B2 phase has many competing slip systems, including <001>{110} and <111>{112}^37^. Anomalous strengthening is not observed in binary B2 alloys, since the critical resolved shear stresses between competing systems are so different, and only one slip mechanism is usually observed. However, the unusual compositional complexity of the B2 phase in the present study may enable two or more different slip systems to compete. It is a further speculation that sessile dislocation debris may be formed by the transition between these different slip systems.

Other mechanisms for anomalous strengthening involve the influence of cross-kinks or the non-conservative ‘dragging’ of jogs^38^. Both of these mechanisms can occur in materials with similar dislocation structures, where deformation is controlled by the motion of screw dislocations. Initial studies show that screw dislocations are also responsible for plasticity in the RHEA^19,39,40^, and so either of these mechanisms are conceivable in the present case.

The discussion offered here is highly speculative. The details of the deformation mechanisms at room temperature and at 600 °C for this RHEA require further investigation, and is recommended for future study. Additional studies are also suggested at higher temperatures and under tensile loading.

In summary, careful microstructural examination of Al_0.5_NbTa_0.8_Ti_1.5_V_0.2_Zr was carried out in the present study using advanced TEM and APT techniques. The results reveal that the homogenized and slow cooled (~10 °C/min) microstructure (Condition (1)) has an ordered B2 phase as the continuous matrix, interspersed with discrete cuboidal-like pockets of a disordered BCC phase. While this microstructure morphologically resembles the γ (fcc) + γ’ (ordered L1_2_) type microstructure observed in many Ni-base superalloys, it is “inverted” in the sense that the continuous matrix phase is the ordered B2 phase, resulting in limited compressive ductility. The ductility of this alloy was substantially increased via microstructural engineering involving controlled annealing treatments, resulting in a phase inversion. Consequently, the hard, ordered B2 compound that forms the continuous phase in Condition (1) is replaced by the more ductile, disordered, BCC phase in Condition (3). The resultant microstructurally-engineered RHEA not only exhibits high yield strength at room temperature (~1345 MPa) and 600 °C (~1423 MPa), but also substantial compressive ductility at room temperature (>20%). The microstructurally engineered Condition (3) also shows higher strength and ductility at 600 °C relative to room temperature – very few systems provide this capability. This opens up the door for the possible use of these RHEAs in real engineering applications. Additional studies are required to further explore these features.

## Methods

### Sample Preparation

The Al_0.5_NbTa_0.8_Ti_1.5_V_0.2_Zr alloy was prepared by vacuum/argon arc melting using high purity (99.9% or higher) elements. Details of the alloy preparation are given elsewhere^9^. The actual composition of the alloy is given in Table 2. After solidification on a water-cooled copper hearth, the alloy was hot iso-statically pressed (HIP’d) at 1200 °C for 2 hours under a super-high purity argon pressure of 210 MPa. After that it was homogenized by holding at 1200 °C for 24 h in a tube furnace with a continuous flow of high purity argon and then slow cooled at 10 °C/min. This condition is called Condition (1). A piece of the sample in Condition (1) was sealed in a quartz tube filled with high purity argon and additionally annealed at 1400 °C for 20 min and water quenched. This condition is called Condition (2). Another piece of the sample in Condition (2) was also sealed in a quartz tube filled with high purity argon and additionally annealed at 600 °C for 120 hrs and water quenched. This condition is called Condition (3).

**Table 2:** Composition of the alloy (in at.%) determined using SEM-EDS.

Components	Al	Nb	Ta	Ti	V	Zr
SEM-EDS	11.3	22.3	13.1	27.9	4.5	20.9

### Characterization

Microstructural characterization was performed using back-scattered electron (BSE) imaging in FEI Nova-NanoSEM 230^TM^ and transmission electron microscopy (TEM) using an FEI Technai G^2^ TF20^TM^ operating at 200 kV. Chemical composition of the alloy, phases and local regions was determined using energy dispersive spectroscopy (SEM-EDS) and atom probe tomography (APT). Atom probe experiments were performed using a local electrode atom probe (LEAP 3000x HR^®^) from Cameca Inc. The TEM foils and the atom probe tips were prepared using Focused Ion Beam (FIB) (FEI Nova 200 NanoLab). All atom probe experiments were conducted in the temperature range of 40–60K, using a voltage evaporation mode with 0.5–1.0% evaporation rate and pulsing voltage maintained to 20% of the steady-state voltage at a frequency of 200 kHz. Raw data from the atom probe experiments were analyzed using Cameca’s IVAS 3.6.8^®^ (Integrated Visualization and Analysis Software).

### Mechanical Testing

Vickers microhardness was measured on polished cross-section surfaces by applying a load of 4.9 N for 15 seconds, and an average of 10 indents were taken into account and the standard deviation in these values has been included in the reported error bars.

The compression samples were extracted from heat treated blanks and surface-polished with 600-grit sandpaper. The final sample dimensions were 4.25 mm × 4.25 mm in the cross-section and 6.8 mm in height, providing the height-to-width aspect ratio 1.6, in agreement with the ASTM standard^41^ and ASM recommendations^42,43^.

Compression tests at 600 °C were conducted using a computer controlled Instron mechanical testing machine outfitted with a Brew vacuum furnace. Silicon carbide dies were coated with boron nitride powder to reduce friction between samples and die contact surfaces. Prior to each test, the furnace chamber was evacuated to 10^−6^ torr, and this or higher vacuum was maintained during testing. The sample was then heated to the test temperature at 50 °C/min, soaked at the temperature for 15 min under 20 N force control, and then compressed at a constant ram speed that provided an initial strain rate of 0.001 s^−1^. Room temperature compression tests were conducted in air using a servo-hydraulic MTS machine at the same strain rate conditions. Thin (~50 μm) Teflon foil was used as a lubricating material between the dies and sample contacting surfaces. Displacement in all tests was monitored and synced with load output from the test frames using Image Correlation software. The compression behavior was analyzed in terms of true stresses and true strains, which take into account a continuous increase in the sample cross-section during compression deformation.

## Electronic supplementary material

Supplementary Figure
