# Supplementary material for: Microstructural Design for Improving Ductility of An Initially Brittle Refractory High Entropy Alloy
Source: Sci Rep. 2018 Jun 11;8:8816. doi: 10.1038/s41598-018-27144-3 (PMC5995863; doi:10.1038/s41598-018-27144-3)
Supplement: Supplementary file 1 — Supplementary Figure [file 41598_2018_27144_MOESM1_ESM.pdf]

Microstructural Design for Improving Ductility of An Initially Brittle Refractory High Entropy Alloy

V. Soni [a,b], O.N. Senkov [c,d], B. Gwalani [a,b], D. B. Miracle [c], and R. Banerjee [a,b] *

[a]Department of Materials Science and Engineering, University of North Texas
Denton, TX-76207, USA
[b]Advanced Materials and Manufacturing Processes Institute, University of North Texas
Denton, TX-76207, USA
[c] Air Force Research Laboratory, Materials and Manufacturing Directorate,

Wright-Patterson AFB, OH-45433, USA

[d] UES Inc., 4401 Dayton-Xenia Road, Beavercreek, OH, USA

Supplementary Figures

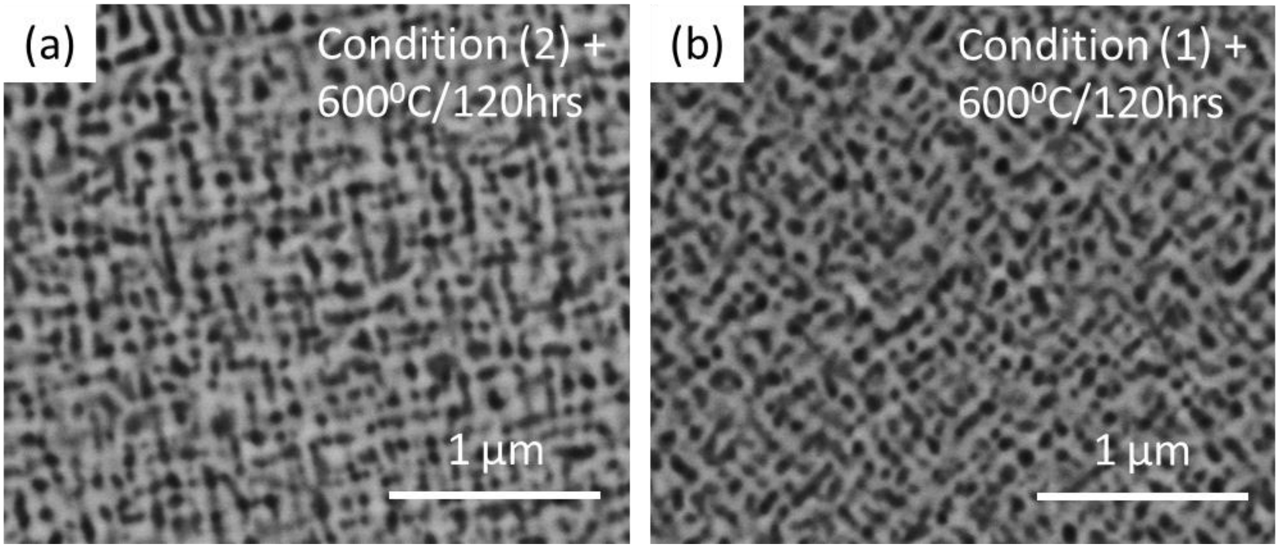

Figure S1. Backscatter SEM images showing the comparison between the microstructure observed after annealing at 600^0C for 120hrs, starting from an initial microstructure of (a) condition (2), and (b) condition (1). No significant differences observed in terms of the size scale, distribution, number density and phase fraction of the B2 precipitates in the BCC matrix. Therefore the microstructure in condition (1) can be considered as an early stage of the microstructure in condition (3).
